# Issues and current standards of controls in microbiome research

**DOI:** 10.1093/femsec/fiz045

**Published:** 2019-04-09

**Authors:** Bastian V H Hornung, Romy D Zwittink, Ed J Kuijper

**Affiliations:** 1 Department of Medical Microbiology, Leiden University Medical Center, PO Box 9600, 2300RC, Leiden, The Netherlands; 2 Center for Microbiome Analyses and Therapeutics, Leiden University Medical Center, PO Box 9600, 2300RC, Leiden, The Netherlands; 3 Netherlands Donor Feces Bank, Leiden University Medical Center, PO Box 9600, 2300RC, Leiden, The Netherlands

**Keywords:** positive control, negative control, microbiome, microbiota, metagenomics, best practices, contamination

## Abstract

Good scientific practice is important in all areas of science. In recent years this has gained more and more attention, especially considering the ‘scientific reproducibility crisis’. While most researchers are aware of the issues with good scientific practice, not all of these issues are necessarily clear, and the details can be very complicated. For many years it has been accepted to perform and publish sequencing based microbiome studies without including proper controls. Although in recent years more scientists realize the necessity of implementing controls, this poses a problem due to the complexity of the field. Another concern is the inability to properly interpret the information gained from controls in microbiome studies. Here, we will discuss these issues and provide a comprehensive overview of problematic points regarding controls in microbiome research, and of the current standards in this area.

## Teaser

Current issues and standards with positive and negative controls in microbiome research are discussed.

## INTRODUCTION

The microbiome field is a relatively new field of research. The hallmark publication by (Venter *et al*. 2004) is not even 15 years old. It was, and still is, exciting to sequence the microbial community of an environment to its full extent, and to learn about all its inhabitants and their possible functions. The subsequent rise in interest in this field was tremendous, leading to numerous publications in highly respected journals, e.g. (Tringe *et al*. 2005; Turnbaugh *et al*. 2009; van Nood *et al*. 2013).

The use of shotgun sequencing or targeted DNA amplicon sequencing to characterize the microbiome has its complications though. Most researchers are aware of the tremendous impact that DNA extraction has on the outcome of any microbiome study (Costea *et al*. 2017; Sinha *et al*. 2017a). It is also clear that these various DNA extraction methods might not produce very overlapping results (e.g. (Angelakis *et al*. 2016), among many others), which makes their interpretation difficult. There are various other concerns which are not specific to this field, but also apply here, like the crisis in reproducibility (Schloss 2018), or the public availability of data in a FAIR (Findable, Accessible, Interoperable and Reusable) way (Langille, Ravel and Fricke 2018).

One particular problem exists, which has been overlooked for a long time: the lack of controls in microbiome research. It is good practice to perform experiments with controls, to ensure that all procedures were correctly performed, and that none of the steps have introduced false positive or false negative results. In microbiome research, however, controls have not been included in the majority of published studies. To clarify the extent of this, we reviewed all publications from the 2018 issues of Microbiome and the ISME journal (manually, as well as keyword searches for ‘mock’, ‘blank’ and ‘control’). From the 265 publications utilizing high-throughput community sequencing of any type (16S, metagenomics, metatranscriptomics, 18S, ITS, virome, PacBio, Nanopore, others), only 30% (79) reported using any type of negative control, and only 10% (27) reported using a positive control. In some of these cases, however, it was unclear if the negative controls were actually sequenced (e.g. when samples were also used for qPCR), or the descriptions were insufficient to judge whether controls were adequate (e.g. ‘appropriate controls were used at all steps’, without further details or ‘methods have previously been validated with positive controls’). During our research we also still noticed several high impact publications, which report results which are potentially indistinguishable from contaminations. Common to all these publications is the lack of controls, paired with the investigation of relatively low biomass microbiomes like mucosa (Zuo *et al*. 2019), amniotic fluid (Wang *et al*. 2018) (despite being heavily discussed earlier (Lauder *et al*. 2016)), gastric environment (Ferreira *et al*. 2018), pancreatic cancer (Pushalkar *et al*. 2018), respiratory tract (Nicola *et al*. 2017), human milk (Drago *et al*. 2017) (although the authors report a clean negative control) and sometimes pure in-silico studies might identify contaminations as biologically relevant (Tackmann *et al*. 2018). These examples come in addition to further publications mentioned in (de Goffau *et al*. 2018), which also did not use negative controls in their study setups. The substantiality of microbiome studies without controls might have been due to a combination of lack of knowledge, unavailability of positive controls and perceived costs associated with the inclusion of non-biological control samples. Indeed, there are numerous challenges with both types of controls in microbiome research, which will be summarized here, together with current developments in this field.

## POSITIVE CONTROLS IN MICROBIOME RESEARCH

### Selection of organisms

For a long time, positive controls were not used in microbiome research due to their unavailability. Positive controls are now commercially available in the form of defined synthetic communities, but their validity for specific research questions is uncertain, depending on the exact microbiome under investigation. For example, the controls from BEI resource, https://www.beiresources.org/Catalog.aspx?f_instockflag=In±Stock%23∼%23Discontinued%23∼%23Temporarily±Out±of±Stock&q=mock%20community, and ATCC, https://www.lgcstandards-atcc.org/en/Products/Cells_and_Microorganisms/By_Focus_Area/Microbiome_Research/Mock_Microbial_Communities.aspx, contain only bacteria, while the ZymoResearch control, https://www.zymoresearch.eu/products/microbiomics, contains bacteria and fungi. Though the manufacturers took care to select relevant bacterial species, including pathogens and Gram-negative and -positive bacteria, it needs to be considered whether such a control is a valid representative for the investigated environment since archaea, viruses and other eukaryotes are not included. Therefore, the presence of archaea, viruses and other eukaryotes might be overlooked in the investigated samples (Bakker 2018). These microbes also pose their own challenges like variable amplicon length and within-species divergence (Palmer *et al*. 2018). New bacterial phyla are also still being discovered, sometimes even multiple phyla with various representatives at once (Karst *et al*. 2018), and we do not know anything about their physiology, including how resistant these organisms will be to the current DNA extraction methods and how they compare to well-studied organisms used in mock communities. This is not a problem of the controls, but of the microbiome field itself, where many of the microbes in these environments are unknown or uncultured. For a proper positive control, we would actually already need to know what microbes are present in a sample. All of this needs to be considered when selecting a positive control. When commercially available controls are unsuitable, custom designed positive controls might be needed, but standardized protocols to do so are currently lacking.

### The interdependency between kits and controls

Another problem is the interdependency between the DNA extraction kit manufacturers and the positive control manufacturers. A kit or a method will be benchmarked using a positive control and this must be a part of the development process of the kit itself. At the end of development, the kit will be able to extract the correct proportions of DNA from the corresponding positive control. Despite the rigorous testing, it cannot be guaranteed that the kit will be able to extract correct DNA proportions from any type of community. Other factors within the community, like physical interactions between different cells or different kinds of metabolites (e.g. glycans) might interfere with the extraction (Angelakis *et al*. 2016), and these factors will vary between communities. This also applies to other potential positive controls, which might have different properties. Using one extraction kit on one mock community will therefore in some cases only indicate its performance on this mock community, and does not necessarily indicate its suitability for real biological samples or even other mock communities. The performance of different kits on varying mock communities has not been tested, and some kits might perform well on some mock communities, and rather poorly on others. The test of one kit with one mock community might therefore not in all cases be sufficient.

### Amplification bias and related errors

If an approach is used which uses PCR amplification during library preparation (including all amplicon technologies), then it also needs to be considered that this step could introduce problems for accurately determining the composition of the microbiome. It has been shown that amplification biases exist, and that DNA fragments with a high or low GC content are not amplified in the same rate as fragments with an average GC content (Aird *et al*. 2011; Benjamini and Speed 2012). The possibly presence of this issue can be discovered with positive controls, since some organisms might be less efficiently amplified. Both ZymoResearch and ATCC offer a pre-extracted DNA mix, which can be used to verify sequencing-related procedures, e.g. library preparation (Jones *et al*. 2015).This will help the researcher to distinguish amplification bias issues from DNA extraction issues (Costea *et al*. 2017; Sinha *et al*. 2017a), but can also give insight into issues related to using varying amounts of DNA for amplification (Bowers *et al*. 2015). Furthermore the various sequencing machines used in the field display different kinds of sequencing errors (Minoche, Dohm and Himmelbauer 2011), which can be specific to the sequenced DNA (Nakamura *et al*. 2011). Errors related to sequencing might furthermore appear randomly, without being reproducible (Yeh *et al*. 2018). This error might lead to the same faulty interpretation like amplification bias, which is that some bacteria might be absent in the sequenced samples, or less abundant than they are. Usage of a positive control will prevent this issue.

### Influence of bioinformatics processing—clustering and filtering

Bioinformatics processing of the sequencing data also contributes to the issues of accurately determining community composition. There are no fully agreed upon standards for raw data processing, and the various parameters of 16s rRNA gene sequence analysis software, like QIIME (Caporaso *et al*. 2010), often need to be tweaked. Ideally, positive controls in microbiome research aid in correct data processing and the parameters can be optimized with working positive controls. A frequently considered parameter is the OTU similarity level for clustering, e.g. 97%, 98.5% or 100% (Patin *et al*. 2013). But any type of clustering based on a similarity of less than 100% might lump two sequences that differ by at least one nucleotide into a single OTU and produce inaccurate results (He *et al*. 2015). Not clustering sequences is not necessarily the solution to this problem, since there can be heterogeneity within the rRNA genes in the same microorganism, and this and other effects might inflate the number of OTUs detected (Nguyen *et al*. 2016; Nearing *et al*. 2018).

### Influence of bioinformatics processing—taxonomic assignment and binning

Usage of a positive control will furthermore enable the researcher to pinpoint a limited amount of possible issues in the taxonomic assignment. As it is known, the public sequence databases contain various errors, including contaminations (Sheik *et al*. 2018), and sequences with incorrectly assigned taxonomy or incorrectly assigned names (Nilsson *et al*. 2006; Federhen 2015). If such an error falls into the taxonomic range of the positive control, the taxonomic assignment might come back faulty. Since the content of the control is known, the researcher might be able to find the sequences which obstruct the correct assignment, and remove these from their database. The general occurrence of this error is not likely, since many of the used pipelines for the assignment of taxonomy based on 16S will discard assignments which are not predominant. In case of metagenomics, this case could be less clear. The full genomic diversity of a species is often not sampled, with only a few specimen being sequenced, and one misassigned species might disturb the taxonomic assignment severely.

Another issue related to this topic is the potential depth of assignment. In some circumstances it is not possible to assign sequences to the correct genus or species, e.g. when genera are too closely related (e.g. *Escherichia* and *Shigella*). If this is the case for some species in the positive control, the researcher will get assignments on e.g. the respective family level, and will be made aware of this restriction and can interpret his data with this in mind. In the case of 16S amplicon data, most researchers will be aware that e.g. their assignment in the family of Enterobacteriaceae might be their specific organism of interest like *E. coli*, but for metagenomics, this problem can be more prevalent. A positive control can obviously aid here only in a limited way, but it makes a researcher potentially aware of this issue.

If binning (Sangwan, Xia and Gilbert 2016) of a genome will be attempted in a metagenomic sample, then it needs to be also considered that a different type of positive control might be more applicable. Having multiple more strongly related organisms (e.g. two strains of the same species) of different abundances in a positive control would be beneficial to in-depth confirm the result of the binning process, especially when more organism-specific parameters like GC content are one of the factors used during the binning process.

## NEGATIVE CONTROLS IN MICROBIOME RESEARCH

Negative controls in microbiome research face also different problems. Some of these are the same as for the positive controls. E.g. it needs to be considered at which step which control is necessary, and which way to sample these is the most suitable. The details of the most important steps will be described below.

### Sampling controls

The first step in which negative controls should be taken into account is at sampling. If a cohort of patients is sampled from a similar site (such as oral swabs) by a trained researcher then a chance exists that either the researcher, the used sampling equipment or the environment could contaminate the samples. Including an appropriate negative control is, however, not always easy. In this setting, a control swab could easily be taken, unpacked in the same surrounding and handled by the same researcher without taking any sample. But the question arises how a negative control would be taken if for example faecal material is considered, since no material to perform a negative DNA extraction would be available, and sampling the air or container with any other kind of instrument like a swab would not be a true negative control. Being consistent when taking samples (Vandeputte *et al*. 2017) is therefore key to minimize technical variation.

### Contamination by the researcher

One of the potential sources of contamination, the researcher itself, also needs to be taken into account. With large numbers of samples, we cannot assume that potential contamination by the researcher will be evenly distributed over all samples during processing. If *Cutibacterium* (formerly *Propionibacterium*) *acnes* (a common skin commensal) unexpectedly appears in samples, but not in the negative controls, it cannot necessarily be concluded that it is not a contaminant from the sample processing, since the possibility exists that only some samples in a bigger cohort are contaminated. This could be resolved by culturing the suspicious organisms from the original sample, but specific bacterial species may be difficult to culture (Staley and Konopka 1985; Rinke *et al*. 2013; Boers *et al*. 2018) from samples containing many different bacterial species. Therefore, a negative culture would not give us definitive proof of absence. Furthermore it does not exclude that the contamination occurred in the original sample, and not during processing.

### The ‘kitome’

The most influential discovery regarding the necessity of including negative controls, is the recognition of a ‘kitome’ (Salter *et al*. 2014). It has become apparent that various DNA extraction kits contain their own unique microbiome, which may be indistinguishable from the real microbiome (e.g. often discussed (Bhatt *et al*. 2013) and mentioned in (Salter *et al*. 2014), as well as multiple examples in (de Goffau *et al*. 2018)). This means that even if the researcher in the laboratory has worked in a complete sterile environment with appropriate techniques, outcomes are affected by the DNA in the extraction kit. While this has been studied extensively, and specific organisms have been identified as common kit contaminants (Laurence, Hatzis and Brash 2014), this might cause problems when environments are studied where these organisms could be present (as mentioned in Jousselin *et al*. 2016), or where it is unknown if they could be present. While these extraction kits are not sold as ‘sterile’, it should also be noted that even if material is sold as ‘sterile’, it might still contain bacterial DNA, and therefore cannot be excluded as a source of contamination (van der Horst *et al*. 2013). Therefore extraction controls need to be performed, but index hopping (see further below) might interfere with this procedure.

### Index hopping

Index hopping is another problem in microbiome research (Costello *et al*. 2018; MacConaill *et al*. 2018), as well as in other fields (Sinha *et al*. 2017b; Griffiths *et al*. 2018). It can occur 0%–10% of the sequenced data (Sinha *et al*. 2017a), depending on the used Illumina platform. If samples are multiplexed during the same run, the possibility exists that indexes from one sample will be incorrectly assigned to another sample. This is caused by non-ligated adapters from one sample (possible a low-biomass sample, where more adapters were in the sample than actual DNA), which will randomly ligate to free DNA from another sample on the same sequencing run. Negative controls might therefore contain data, which incorrectly originates from the other samples during the same sequencing run (although not always, since this can depend on the way the samples are loaded onto the sequencer). In practice, the negative controls might contain exactly the same profile as the sequenced samples. In these cases it is impossible to distinguish between true contamination and index hopping, making the controls (negative as well as positive) potentially useless. Also, proposed practices like the subtraction of shared OTUs between negative controls and samples (Edmonds and Williams 2017) would falsify the results in this situation, since the contamination is derived from the samples, and not from the researcher, DNA extraction kit, or the environment. This could be circumvented by sequencing negative controls on a separate run, but again, in practice this will increase the price of sequencing to an unacceptable high level, and is therefore highly unlikely to happen. If the sequencing is not performed in-house, this would also complicate the procedure even more, since it would be necessary to request different lanes for different samples. The final result would also no longer be a control of the whole process, since samples will not be sequenced at the same time and on the same machine anymore. While evaluating a sample, it therefore needs to be considered if data in a low biomass sample and negative controls could be derived from a high biomass sample or positive control, and results need to be interpreted with caution.

## CURRENT STANDARDS AND DEVELOPMENTS

No easy solutions exist for many of the above mentioned problems. It can be advised to take negative controls during the sampling process and at all further steps, if feasible (for example Galan *et al*. 2016; Jousselin *et al*. 2016; Zhong *et al*. 2018). New methodologies, that reduce the amount of contamination during processing, might also be necessary, e.g. (Boers, Hays and Jansen 2017; Minich *et al*. 2018), but their implementation in the laboratory is not always easy.

For the data analysis of negative controls it is mainly advised to focus on the number of reads obtained. A clean negative control should have few reads, which excludes major contaminations from all possible sources. Kitome components or actual contaminations in these negative controls are in such cases unlikely to be abundant, and unlikely to have a significant impact on the analysis, even if present. The outcome of the negative controls are particularly relevant for samples with low microbial biomass (Biesbroek *et al*. 2012; Lusk 2014; Glassing *et al*. 2016; Karstens *et al*. 2018; Velasquez-Mejia, de la Cuesta-Zuluaga and Escobar 2018), since even low amounts of contamination could have an impact here. Especially in these cases the connection between the samples and the corresponding negative controls needs to be carefully evaluated. A possibility to resolve this contamination is to remove OTUs identified in the negative controls from the actual samples (e.g. Edmonds and Williams 2017). This is only applicable, when it can be ensured that these are actually contaminants, and not e.g. derived from another sample, as explained in the index hopping section. If a positive control in the same run has potentially acquired contaminations, then this would help in resolving this issue partially. Additional low abundance OTUs in the positive controls will give the researcher an idea which level of minor abundances can be reasonably filtered out. This again applies mainly for samples with a high biomass, while for low biomass samples more caution is needed.

Further developments to prevent index hopping by usage of multiple indexes at the same time (Costello *et al*. 2018; MacConaill *et al*. 2018) will hopefully make this interpretation easier in the future, since then it would be possible to also exclude contamination from high biomass samples on a run with low biomass samples. Currently this is not yet the case, which makes resolving the source of contamination often complicated.

The observation that the concentration of contaminants is inversely correlated to amplicon concentration has been reported multiple times (Salter *et al*. 2014; Jervis-Bardy *et al*. 2015; Lazarevic *et al*. 2016). While it has not yet been implemented, the microbiome research community might need to consider using a dilution series of single samples as a control, to make the identification of contaminants easier, although specific challenges also apply (Multinu *et al*. 2018). Standards need to be agreed upon, since having different dilution ratios might make the comparison between studies complicated. The dilution steps will most likely be dependent on the expected biomass, and a general protocol how to determine the appropriate steps will be necessary. Software solutions to address the outcome of such negative controls have already been developed (Davis *et al*. 2018), and seem to be performing well (Karstens *et al*. 2018), so that researchers are directly able to deal with their control samples.

Another indicator of contamination in metagenomic samples can be derived from the insert size of the reads (Olm *et al*. 2017). Olm *et al*. identified a contaminant in their dataset by aberrant insert size distribution, indicating another origin of the DNA. The low heterogeneity within the contaminant data may also be an useful indicator, though it is not applicable in all cases, e.g. when strain transfer between microbiomes is a part of the research question (Smillie *et al*. 2018).

The idea of adding spike-in controls (addition of external microbial DNA to a sample) could be an interesting approach. It will not only allow tracking of samples, but also aid absolute quantification and quantification of cross-contamination/index hopping (Galan *et al*. 2016; Hardwick *et al*. 2018; Palmer *et al*. 2018; Tourlousse, Ohashi and Sekiguchi 2018), and should probably be implemented for low-biomass samples. Furthermore, it seems that some kind of quality control regarding misassigned sequences is even possible after the experiment has been conducted (Wright and Vetsigian 2016), but as the authors point out, this might need to be investigated on a per experiment basis, and therefore does not seem to be very practical in most circumstances. Another computational method to correct for misassigned sequences has been developed for single-cell data (Larsson *et al*. 2018), but its potential applicability to metagenomics data still needs to be shown.

For positive controls, the most diverse mock community available should be chosen, if it is applicable for the proposed research project. This should minimally prevent overfitting of a protocol to the used control, although it does not deal with the microbial dark matter. The possibility of creating one's own mock community can be considered, in case these samples will be processed more often and in case the available mock communities are not enough comparable to the investigated microbiome.

If expected organisms do not appear in the mock community, then a PCR for identifying species specific genes in the mock community needs to be performed, to ensure that this organism was actually present in the sequenced sample. The same applies for unexpected occurrences of contamination in the mock community.

In general, for good scientific practice, controls should be included at all steps. This should be done with the consideration that the microbiome field is developing rapidly and during the progress of a research project new methods might be published, which will help with proper interpretation. Even if proper interpretation is not possible, publishing the results from the positive and negative controls needs to be mandatory, so that the reader can interpret the findings with caution if necessary.

## CONCLUSIONS

While the field of microbiome studies has become an established research area, and many best practices exist (Goodrich *et al*. 2014; Boers, Jansen and Hays 2016; Kim *et al*. 2017; Knight *et al*. 2018; Martin *et al*. 2018; Pollock *et al*. 2018), they have not gone in-depth regarding the usage of controls. We summarized the issues regarding good scientific practice in light of controls, to make scientists aware of these issues, so that they can be addressed in their own research. Many of the problems regarding the lack of controls have been recognized very recently, many even last year, and new strategies to prevent these are likely to be developed soon. While no standards exist for the processing and interpretation of controls, any further studies in this field need to include controls to prevent erroneous results and improve data interpretation. We therefore urge that future study designs must include all the necessary controls, as listed below, until the scientific community has agreed upon better standards:

- A negative control for sampling, to ensure that sampling equipment (tubes, swaps, etc) are not contaminated.
- A negative control for DNA extractions, to ensure identification of potential kit contaminations.
- A negative control for sequencing, to ensure that no large-scale cross-contamination between samples takes place.
- A positive control for DNA extraction, to ensure that the contained organisms can be sufficiently extracted with the used methods.
- A positive control for sequencing (a pre-extracted DNA mix), to ensure that the sequencing itself did not introduce any errors.

We furthermore would like to appeal to the journals in the microbiome field to introduce a screening procedure/checklist for these items, e.g. some journals have done to ensure proper replication within their publications.
